# Defining Hypoperfusion in Chronic Aphasia: An Individualized Thresholding Approach

**DOI:** 10.3390/brainsci11040491

**Published:** 2021-04-13

**Authors:** Noelle T. Abbott, Carolyn J. Baker, Conan Chen, Thomas T. Liu, Tracy E. Love

**Affiliations:** 1 San Diego State University and University of California San Diego Joint Doctoral Program in Language and Communicative Disorders, San Diego, CA 92182, USA; cjbaker@ucsd.edu (C.J.B.); tlove@sdsu.edu (T.E.L.); 2 Center for Functional MRI and Department of Radiology, University of California San Diego, San Diego, CA 92093, USA; coc004@health.ucsd.edu (C.C.); ttliu@health.ucsd.edu (T.T.L.); 3 School of Speech, Language, and Hearing Sciences, San Diego State University, San Diego, CA 92182, USA

**Keywords:** chronic aphasia, stroke, cerebral blood flow, hypoperfusion, perfusion imaging, perilesional tissue, language behavior, individual differences

## Abstract

Within the aphasia literature, it is common to link location of lesioned brain tissue to specific patterns of language impairment. This has provided valuable insight into the relationship between brain structure and function, but it does not capture important underlying alterations in function of regions that remain structurally intact. Research has demonstrated that in the chronic stage of aphasia, variable patterns of reduced cerebral blood flow (CBF; hypoperfusion) in structurally intact regions of the brain contribute to persisting language impairments. However, one consistent issue in this literature is a lack of clear consensus on how to define hypoperfusion, which may lead to over- or underestimation of tissue functionality. In the current study, we conducted an exploratory analysis in six individuals with chronic aphasia (>1 year post-onset) using perfusion imaging to (1) suggest a new, individualized metric for defining hypoperfusion; (2) identify the extent of hypoperfused tissue in perilesional bands; and (3) explore the relationship between hypoperfusion and language impairment. Results indicated that our individualized metric for defining hypoperfusion provided greater precision when identifying functionally impaired tissue and its effects on language function in chronic aphasia. These results have important implications for intervention approaches that target intact (or impaired) brain tissue.

## 1. Introduction

Aphasia is a language impairment that typically results from neurological injury to the perisylvian language zone in the language-dominant hemisphere of the brain (generally the left hemisphere). Aphasia can include disturbances of production and/or comprehension skills for spoken, written and/or signed language modalities. Aphasia is a pervasive disorder, with at least 2 million people in the USA currently living with the disease [1]. Disruption of language abilities has been linked to high levels of depression, poor social outcomes and a reduction in autonomy [2]; and has also been linked to greater levels of stress for caretakers of post-stroke individuals [3]. Given the enormous impact that aphasia has on quality of life for all of those affected, treatment approaches should be aimed at maximizing recovery to promote better outcomes for these individuals. One way to do so is to bootstrap recovery by taking advantage of intact neural mechanisms. This involves understanding both the structural and functional integrity of the brain-language network post-stroke. In this paper, we focus on one measure of brain function, cerebral blood flow (CBF), within structurally intact brain regions to understand how it supports language function and how it can be used to better inform novel treatment approaches in the chronic stage of aphasia.

Since the 1800s, scientists such as Paul Broca and Karl Wernicke have sought to characterize the relationship between different brain structures and their functions by mapping out how damage to a specific brain region alters behavior. However, one of the challenges to this localizationist approach has been that following a stroke, individuals with aphasia (IWA) often demonstrate variable patterns of language impairment and recovery [4,5]. With the advancement of neuroimaging techniques, structure-function relationships have been investigated with more sophisticated methods, such as voxel-based lesion-symptom mapping (VLSM) [6]. Although VLSM has begun to elucidate the role of specific brain regions in language, important individual differences in brain organization and function may be missed by only considering focal lesion damage in isolation, as it assumes normal neurophysiological function in structurally intact brain regions. Indeed, research in aphasia has found that while both lesion size and location are important factors in predicting language behavior post-stroke, factors such as language reorganization, the functional integrity of connected regions, and alterations in vascular physiology contribute to the recruitment of neural tissue [7,8,9]. Thus, studies that rely solely on structural brain mapping techniques to investigate isolated regions of a behavioral network will inevitably miss functional changes, such as when physical damage to one region of the brain results in disruptions of CBF both near and distal to the lesion, a process known as diaschisis [10,11,12]. This missing part of the picture is argued to underlie the variability that we see in linking brain and behavior. We believe one way to account for functional brain damage (as opposed to structural) is through the use of perfusion imaging.

### 1.1. Perfusion

Perfusion refers to the delivery of blood to neural tissue and can be quantified by measuring the amount of blood delivered to a given amount of tissue per unit of time (mL/100 g of tissue/min) [13]. There is a tight coupling between CBF and neuronal activity (e.g., neurovascular coupling) [14]. When blood is perfused throughout the brain, it delivers nutrients such as oxygen and glucose to neural tissue. When a neuron fires, blood flow to that region increases so that it has enough fuel to perform its specified function [15]. This increase in CBF underlies the methods historically used to gauge a region’s engagement in behavioral tasks (e.g., fMRI). If CBF to neural tissue is disrupted in any way, it will impact neuronal function, which will ultimately affect behavior.

Throughout the literature, scientists have quantified the amount of CBF needed to sustain neuronal activity. In doing so, these quantified values (described below) have been assumed to capture functionality across individuals. Unfortunately, this standard approach may over- or underestimate the functional integrity of structurally intact neural tissue. Much like individuals demonstrate variability in behavior post-stroke, they also demonstrate variability in how much blood flows to a brain region over a specified time interval. It is these individualized patterns that are key when attempting to link brain and behavior.

CBF ranges change over the lifespan but in general, for unimpaired adults, normal CBF is between 50 and 60 mL of blood/100 g of tissue/min in gray matter [15,16,17]. According to standard cutoff values, when blood flow falls below 20 mL/100 g of tissue/min, oxygen levels become too low to support optimal neuronal metabolism, leading to impacts on cognitive function [18,19]. As CBF falls below 12 mL of blood/100 g of tissue/min, neurons become oxygen deprived and die, resulting in necrotic brain tissue. When regional baseline CBF levels are adequate enough to sustain neural tissue viability but inadequate for efficient brain function (i.e., within the range 12–20 mL of blood/100 g of tissue/min), they are referred to as hypoperfused, or in other words, are functionally rather than structurally lesioned. Following a stroke, hypoperfusion is commonly seen in regions proximal to the lesion, but prior research has also found that regions distal to the ischemic core may be hypoperfused [20,21,22]. For IWA, this suggests that hypoperfusion may contribute to language impairments that would not otherwise be predicted by structural brain damage alone.

### 1.2. Hypoperfusion in Aphasia

The majority of research looking at hypoperfusion in aphasia has focused on the acute stages of stroke, with far less work focusing on the impacts of reduced CBF in chronic stroke (>1 year post-onset). Unlike in acute stroke, studies looking at the role of hypoperfusion in chronic stroke have largely focused on study defined perilesional (i.e., penumbra) tissue, with limited work extending beyond those boundaries. The sparse evidence investigating hypoperfusion in left hemisphere regions outside of the core lesion has suggested that it may provide insight into persisting language impairments and functional recovery in chronic aphasia [12,20,22,23,24,25].

In a case study investigating the neural source of reading impairment in a participant with chronic aphasia, Love et al. (2002) found hypoperfusion in two brain areas associated with reading, the angular gyrus and supramarginal gyrus. Interestingly, neither of these regions appeared structurally damaged on an MRI scan suggesting that the impairments demonstrated were due to functional rather than structural brain damage [24]. Similarly, Brumm et al. (2010) found areas of regional hypoperfusion in structurally intact regions in their group of chronic IWA and importantly, demonstrated that the inflow curve (the time it takes CBF to perfuse throughout the brain) may be delayed in individuals with chronic aphasia [20]. This timing of CBF is critical when applying methods that rely on measuring the hemodynamic response during particular behavioral tasks and may be the reason for some discrepant findings in the literature. These patterns have been further demonstrated in a recent study by Robson et al. (2017) where the investigators found that hypoperfused regions outside of core lesions were significantly correlated with behavioral language impairment [12]. These studies suggest that functional brain damage can account for language impairments that would not otherwise be predicted on the basis of structural damage alone and this functional disruption likely introduces variability when attempting to model the roles of specific brain areas in language function.

In addition to remote regional hypoperfusion in chronic aphasia, there also seems to be a correlation between language impairment and CBF in the perilesional tissue surrounding the core lesion, as has been found in acute stroke. As we turn our focus to perilesional tissue in chronic aphasia, we must first point out that there is no clear standard on how to define the extent of perilesional tissue. Early evidence has defined perilesional tissue as extending anywhere from 0 to 15 mm beyond the lesion’s border. Richardson et al. (2011) found that perilesional tissue between 3 and 8 mm from the lesion’s rim was highly susceptible to hypoperfusion [25]. Similarly, Thompson et al. (2017) found a correlation between language impairment and CBF values in perilesional tissue extending 0–6 mm beyond the lesion’s border, with lower values indicating greater impairment [22]. However, they did not find a correlation between CBF values in language regions of interest (ROIs) outside of perilesional tissue and behavioral impairments in the chronic stage. In another study by Fridriksson et al. (2012), the authors explored functional activation changes within perilesional tissue before and after an Anomia treatment protocol and found that increased activation in perilesional tissue was related to increased performance in naming abilities [26]. Interestingly, in this study, the authors attempted to define perilesional tissue based on CBF values and determined that in their sample of patients, functional damage extended up to 15 mm beyond the lesion’s border.

Across all of these studies that attempt to identify perilesional tissue, hypoperfusion is not defined based on thresholded values, but instead is based on group-level analyses that explore whether there are significant differences between the affected hemisphere and right hemisphere counterparts. When there is a significant change in CBF from one perilesional ring to the next or when perilesional CBF is similar to CBF in the right hemisphere homologue, it is assumed that the difference indicates an overall change in functionality. However, this may be faulty logic. CBF values, especially post-stroke, are known to vary widely among individuals as a result of autoregulatory processes, vascular changes and age [22,27]. While employing group-level analyses to understand how blood flow impacts language behavior is important, this approach may overlook important individual patterns that can affect the interpretation of how different regions support language function. Below we present an alternative approach in identifying hypoperfused regions of the brain. To foreshadow, by determining individualized thresholds of CBF, we argue that we can better identify functionally compromised tissue, which will allow us to individually map when perilesional tissue becomes “normal” and better account for language behavior in chronic aphasia.

### 1.3. The Role of Perilesional Tissue in Chronic Stroke

In order to best understand how to characterize tissue in and around the lesion in the chronic stage, we must first look at how affected tissue is defined during the acute stages of stroke. In the acute stage, immediately after the stroke, there are three identifiable regions of affected brain tissue: (1) the ischemic core, or necrotic brain tissue that has been irreversibly damaged; (2) the ischemic penumbra, or tissue surrounding the ischemic core that is susceptible to infarction if reperfusion does not occur within a timely manner; and (3) an area of benign oligemia, the area between the unaffected region and the ischemic penumbra [28,29]. In the subacute stage, during the first three to six months after the ischemic core has resolved, the remaining perilesional tissue surrounding the core lesion becomes prone to cellular changes that promote neurogenesis and ultimately lead to reorganization of function [30]. During this stage, there may be functional changes in brain regions connected to the ischemic core and penumbra (i.e., diaschisis) [31]. However, as blood flow surrounding the lesion increases, areas of remote dysfunction begin to resolve [32]. Thus, in the subacute stage, functional brain changes are highly influenced by CBF in regions both proximal and distal to the lesion [30]. Finally, in the chronic stage, the areas directly affected by the stroke include the ischemic core and the perilesional tissue surrounding it (i.e., the ischemic penumbra). Perilesional tissue has been the subject of many studies aimed at recovery of language in the chronic stage as the tissue is functionally compromised but still remains viable [26,33,34,35,36]. Due to the cellular changes and restoration of blood flow that occur in the acute stages of stroke, it is reasonable to believe that the extent of perilesional tissue varies across individuals in the chronic stage and may contribute to variability in behavior.

### 1.4. The Current Study

In this exploratory investigation, we examined whether determining hypoperfusion based on individual patterns better allows for an understanding of neuronal health and functionality in chronic aphasia by (1) first defining hypoperfusion in each of our participants; (2) then using these individualized metrics to determine the extent of hypoperfused tissue in perilesional bands; and (3) exploring how hypoperfusion in language regions of the brain is related to language behavior in chronic aphasia. In doing so, this will allow us to better define when perilesional tissue (which has been the target of many treatment studies) becomes functionally more “normal” and will help us identify functionally affected, but viable tissue in chronic aphasia. We argue that approaching diagnosis and treatment with information on both structural and functional integrity of the brain’s language network can aid translational researchers in developing novel treatment approaches to language impairment as well as clinicians in identifying treatment approaches that take advantage of intact mechanisms to maximize recovery.

## 2. Methods

### 2.1. Participants

All participants were diagnosed with aphasia following a single (first time), left hemisphere ischemic stroke and were in the chronic stage (>4 years post-onset). Participants included 6 individuals with aphasia (IWA; 2 females) with a mean age at testing of 63 years old (range = 55–76 years old). A summary of demographic information is provided in Table 1. There were no reports of pre-morbid neurological or psychiatric disorders, or active or significant drug or alcohol abuse. All participants were native English speakers, had normal or corrected visual acuity and hearing, and were pre-morbidly right-handed. Participants were recruited from San Diego State University (SDSU) and University of California, San Diego (UCSD) and were compensated for their time. This study was approved under both universities’ IRB protocols.

### 2.2. Behavioral Measures

Aphasia subtype, extent and severity were determined based on clinical consensus and standardized language assessments, including the Boston Diagnostic Aphasia Examination, version 3 (BDAE-3) and the Western Aphasia Battery-Revised (WAB-R) [37,38]. The WAB-R is a standardized assessment comprised of multiple cognitive and linguistic subtests. Aphasia diagnosis and an aphasia severity quotient (AQ) were derived from the WAB-R. Lower AQ scores indicate a more severe language impairment. Additionally, we assessed auditory comprehension through the BDAE-3 and WAB-R subtests (BDAE-AC and WAB-AC, respectively; see Table 1 for scores).

## 3. Neuroimaging Analysis

### 3.1. Image Acquisition

High-resolution structural scans were acquired at the UCSD Center for Functional MRI using a General Electric (GE) Discovery MR750 3.0T whole-body scanner equipped with an 8-channel head coil. Anatomical imaging was performed using an inversion prepared fast spoiled gradient-echo (FSPGR) sequence that yielded T_1_-weighted images with 1 mm isotropic resolution. Other parameters were as follows: field of view (FOV) = 25 cm, repetition time (TR) = 8.1, echo time (TE) = 3.1 ms, inversion time (TI) = 450 ms, flip angle 12°, 172 sagittal slices, 256 × 128 matrix, total scan time = 7:30 min.

Resting-state cerebral blood flow (CBF) data were acquired using an optimized pseudo-continuous arterial spin labeling sequence (Opt-PCASL). With this method, the magnetization of protons in the blood is inverted using RF pulses. The inverted magnetization is then used as an endogenous tracer to track blood flow throughout the brain [39]. Unlike the conventional PCASL method, which is sensitive to phase tracking errors that can lead to inaccurate CBF estimates, Opt-PCASL employs an additional prescan procedure to determine optimal RF phase and in-plane gradient amplitudes [40]. Scan parameters were as follows: labeling duration = 1.9 s, post-labeling delay = 2.0 s, two background suppression pulses with inversion times of 1570 ms and 390 ms, TE = 3.2 ms, TR = 4.5 s, slices = 20, slice thickness = 6 mm, FOV = 22 cm, matrix size = 64 × 64, total scan time = 9:54 min.

A time-of-flight angiogram was acquired using a 3D spoiled gradient echo sequence to define the optimal tagging plane location for continuous inversion of arterial blood during the Opt-PCASL sequence described above (FOV = 22 cm, TR = 20 ms, TE = 2.7 ms, flip angle 15°, axial slice thickness = 1.0 mm, 0.43 × 0.43 × 1.0 mm^3^ resolution, total scan time = 1:55 min). The tagging plane was located by visually examining the angiogram to find where the carotid and vertebral arteries were most parallel to one another and perpendicular to the tagging plane. A proton-density weighted scan was acquired to measure the equilibrium magnetization (M_0_), which is used to convert the ASL difference signal into physiological units (i.e., 100 mL of blood/100 g of tissue/min; TR = 4000 ms, TE = 3.3 ms, 9 repetitions with a 90° excitation pulse turned off for the first 8 repetitions, only the 9th image is used; total scan time = 40 s) [41].

### 3.2. MRI Data Processing

#### 3.2.1. T_1_-Weighted Structural Images

For anatomical processing, the T_1_-weighted structural images were registered to the ASL data and segmented into gray matter (GM), white matter (WM), and cerebrospinal fluid (CSF). These steps were performed using tools from Analysis of Functional NeuroImages software (AFNI), and FMRIB Software Library (FSL), respectively [42,43]. Structural images were then manually skull stripped in AFNI by creating individual, hand-drawn brain masks in native space which were used to exclude any voxels outside of brain tissue. This allowed for more accurate results near areas of lesioned tissue. Individual brain hemisphere masks were also created from the whole-brain masks to determine the total volume of each participant’s left and right hemisphere (Table 2). Finally, all structural data were resampled to match the resolution of the ASL data.

To allow us to investigate hypoperfusion within different regions of interest (ROIs), the anatomical images were transformed from native space to the Montreal Neurological Institute (MNI) Colin27 brain by first masking out lesioned brain tissue using manually delineated lesion masks, then using FSL FLIRT to perform affine registration, and finally performing an additional non-linear registration using in-house software. Once transformed, ROIs were labeled using the Automated Anatomical Labeling (AAL) atlas [44]. Linear registration was used to align the AAL atlas with the T_1_ anatomical and CBF images so that we could extract mean CBF values in our ROIs. All values within the AAL ROIs reflect the remaining CBF in intact tissue, as lesioned brain tissue was removed from the analysis. Finally, all structural data were resampled to match the resolution of the ASL data.

#### 3.2.2. Lesion and Perilesional Masks

**Figure 1:**
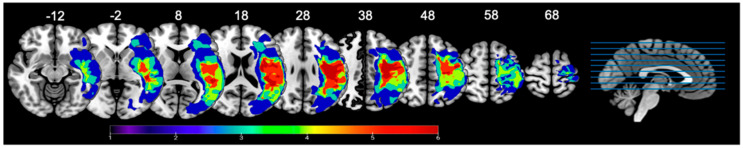
Lesion overlay maps illustrating lesion location and extent in our sample of 6 participants. Heat maps correspond to the number of patients that have a lesion in that area. Lesions are overlaid on labeled axial slices (z-coordinates) from the SPM152 template; SPM: Statistical Parametric Mapping. Images are presented in radiological view (i.e., left hemisphere is on the right side).

To determine the extent of structural brain damage within the left hemisphere, lesion masks were manually created for each participant in native space by delineating damaged brain tissue on the T_1_-weighted images using AFNI (see Figure 1 for lesion overlap map). Total lesion volume was calculated in cubic centimeters (cc^3^) and percent tissue damage in the left hemisphere was calculated by dividing the lesion volume by the total left hemisphere volume (see Table 2). To systematically investigate the perilesional region, four 3-mm (mm) lesion masks were created, one that extended 0–3 mm (3 mm) beyond the lesion’s border, one that extended 3–6 mm (6 mm), another that extended 6–9 mm (9 mm) and a final one that extended 9–12 mm (12 mm). Perilesional masks were then resampled to the resolution of the ASL data and only those voxels that were at least 60% occupied by non-zero, positive values were kept to account for partial voluming effects near the lesion. The original lesion masks and the perilesional masks were flipped to the right hemisphere, to create five additional right hemisphere ROIs (right lesion, right 3 mm perilesional band, right 6 mm perilesional band, right 9 mm perilesional band, and right 12 mm perilesional band). Mean CBF within each ROI was then calculated for each participant.

### 3.3. ASL Data

ASL data were processed using code originally developed for the Cerebral Blood Flow Biomedical Informatics Research Network (CBFBIRN) analysis pipeline [45]. This code included several MATLAB scripts for field map correction and quantification of CBF and utilized AFNI for the volume registration and alignment steps. The ASL data were field map corrected and volume registered, while the M_0_ data were aligned to the ASL data. Finally, the ASL data were quantified into physiological CBF units via local tissue correction with the aligned M_0_ data.

To minimize partial volume effects due to reduced CBF values in white matter or increased volume of CSF within damaged tissue areas [46], we adopted the method described by Johnson et al. (2005) [47]. With this method, CSF is assumed to have a CBF value of 0, while GM is assumed to have a CBF value that is 2.5 times greater than the value in WM. Therefore, to calculate corrected CBF values, we used the following formula:

CBF_corrected_ = CBF_uncorrected_/(f_GM_ + (0.4 × f_WM_))

where f_GM_ and f_WM_ denote the partial volume fractions. To make sure there were no spurious CBF values due to noise, the CBF_corrected_ data were then thresholded to remove any CBF values outside of the range of 0–130 mL blood/100 g tissue/min. This range was selected to allow for the possibility of both hypo- and hyper-perfused tissue. The thresholded CBF maps were then overlaid on top of the segmented gray matter images to create gray matter CBF maps. Importantly, any gray matter voxels that overlapped with the left hemisphere lesion mask were removed. This allowed us to exclude the lesion when calculating CBF within specific ROIs, as values of zero within the lesion lead to substantially smaller CBF values. The gray matter CBF maps were then overlaid on top of the AAL atlas to determine the mean CBF within each ROI for each participant. Importantly, since lesioned tissue was excluded in our calculations, any CBF values of zero were likely the result of partial voluming effects between the gray matter CBF maps and the ROIs. Thus, any voxels with a CBF value of zero were excluded from the mean calculation.

## 4. Results

All analyses were performed using R [48]. For each analysis, areas of lesioned tissue were excluded from the calculations as very low CBF values within the lesion (group level: M = 10.12, SD = 5.79) would substantially lower the left hemisphere mean value (group level: M = 46.94, SD = 16.14).

### 4.1. CBF Comparisons: Left Versus Right Hemisphere

To understand general CBF patterns within gray matter for each participant, we calculated the mean, standard deviation, and median CBF values within the whole brain (global CBF), the left hemisphere (CBF_LH_), and the right hemisphere (CBF_RH_). These metrics were calculated at both the group- and individual- levels (the median was to ensure that average CBF_RH_ was not heavily influenced by extreme values; Table 3). To demonstrate the extent of the differences between CBF_RH_ and CBF_LH_ values, a perfusion ratio was also calculated (CBF_LH_/CBF_RH_); where values < 1 indicate lower CBF_LH_, and values > 1 indicate greater CBF_LH_.

An analysis of variance (ANOVA) showed that at the **group level**, there were significant differences in mean CBF_LH_ (M = 46.94, SD = 16.14) and mean CBF_RH_ (M = 54.92, SD = 16.14), F (1,5) = 17.62, *p* = 0.009, with the left hemisphere having overall lower CBF (perfusion ratio = 0.85). This pattern was similar to what was seen at the individual level (reported below), but individual analyses allowed for a more fine-grained interpretation of differences in CBF between the two hemispheres.

To determine whether there was a difference in mean CBF values between the two hemispheres at the **individual level**, paired t-tests were conducted. Results showed that there were significant differences between left and right hemisphere CBF for all participants except for S03 (as shown in Table 3). For those who had a significant difference, mean CBF_LH_ was less than mean CBF_RH_ as indicated by the perfusion ratios. For S03, the perfusion ratio was 0.95, which indicates that the left hemisphere was lower than the right on average, but this difference was not statistically significant. When comparing the results from the group analysis to the individual analysis, we see that group analysis did not capture the similarity between CBF in the two hemispheres for S03, whereas individual-level analyses not only captured this difference but allowed for a more comprehensive picture of the integrity of the remaining structurally intact left hemisphere.

### 4.2. Lesion Size and CBF

**Figure 2:**
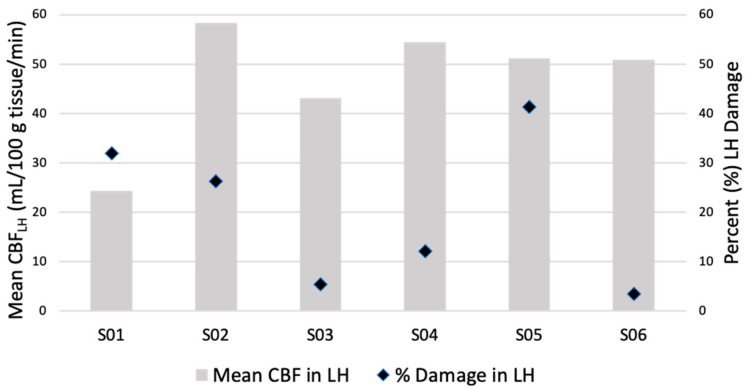
A comparison between mean CBF in remaining intact tissue of the left hemisphere (CBF_LH_) and percent lesion damage in the left hemisphere (LH). There is no clear relationship between mean CBF_LH_ and lesion size in our sample which suggests that CBF in remaining intact tissue is not influenced by lesion size alone.

Although there is a relationship between lesion size, lesion location and language recovery in post-stroke individuals with aphasia, a link between lesion size and overall CBF in the remaining intact tissue of the left hemisphere has not been established [9,49,50]. To directly test whether CBF values are influenced by the amount of structural damage within the left hemisphere, we looked for a correlation between CBF in the remaining left hemisphere (without the lesion) and the percent of lesion damage in the left hemisphere at the group level. Results showed no relationship between CBF in the intact left hemisphere and percent damage, r (4) = −0.19, *p* = 0.72 (Figure 2). Comparisons using t-tests at the individual level illustrate this pattern further. For example, S05, who has the largest lesion in our sample (41.4% lesion damage in the left hemisphere), showed no significant differences in CBF compared to S06 who has the smallest lesion (3.5% lesion damage in the left hemisphere; t (84) = 0.09, *p* = 0.47). S04, who has a medium size lesion in our sample (12.1% lesion damage in the left hemisphere), and S02, who has a large lesion (26.3% lesion damage in the left hemisphere), again showed no significant differences in CBF when compared to one another (t (84) = −1.46, *p* = 0.07). From our current sample, lesion size does not appear to influence CBF values in the remaining intact left hemisphere; in other words, a small lesion does not imply that the remaining tissue will be functional.

Thus far, we have demonstrated that after a stroke, CBF patterns are lower in the remaining tissue of the left hemisphere as compared to the right hemisphere, and this is true at the group level and for the majority of our participants at the individual level. We have also shown that the size of the lesion itself does not impact overall CBF values of the remaining tissue in the left hemisphere. We now turn to the question of how to best define functionally compromised tissue in the remaining intact tissue of the left hemisphere.

### 4.3. Defining Functionally Compromised Brain Tissue

Recall, one of the goals of this paper was to establish individualized thresholds of CBF in chronic aphasia so we can better identify functionally compromised tissue. To accomplish this, we had to determine what constituted “normal” and “functionally compromised” CBF for each participant. Since our participants have no overt evidence of right hemisphere stroke or injury, we chose to use each participant’s average CBF_RH_ as their normal value. We acknowledge here that evidence in the literature has pointed to right hemisphere engagement in certain behaviors following a left hemisphere stroke [51]. We chose to compare CBF_LH_ to average CBF_RH_ so as to capture comparable regions in the non-lesioned hemisphere. In spite of the potential increase in CBF_RH_, we believe that this is the correct contrast as: (1) these were resting-state scans, so theoretically there should not have been behaviorally driven increases in CBF; and (2) in addition to the mean, we also calculated each participant’s median and standard deviation in the right hemisphere (see Table 3) and found no significant differences between the mean and median (t (5) = −1.56, *p* = 0.09).

Our next step after establishing a normal CBF value for each participant was to identify hypoperfused tissue. As discussed previously, there are certain physiological requirements needed for neuronal health and function. One aspect that was missing in the previous discussion was how neuronal function is impacted when CBF values are between 25 and 50 mL/100 g tissue/min. According to a more fine-grained description, when CBF is between 25 and 50 mL/100 g tissue/min, cellular and chemical changes in the brain begin to take place, such as a decline in protein synthesis (~50 mL/100 g tissue/min) and an increase in glutamate (~30 mL/100 g/min), which can trigger the release of destructive enzymes [52,53]. Many of these changes lead to the activation of coordinated activities within the brain to protect the viability of neurons, the vasculature, and other cells and systems [54]. In the chronic stage post-stroke, certain areas of brain tissue may exist within this perpetually compromised state by which tissue remains viable through compensatory mechanisms but is functionally altered. We wish to point out here that there are potential problems with interpreting functionality based on these strict number scales. For example, one of our participants (S03) has an average CBF value for their intact right hemisphere that falls in this potentially compromised range, but their CBF_RH_ does not link directly to impairment as noted by their low severity indices on the BDAE-3 and WAB-AQ (see Table 1). Using the standard cutoff values to identify functionally compromised tissue in this case demonstrates how incorrect conclusions can be made. We therefore argue that it is critical to calculate individualized CBF thresholds to determine functionally compromised tissue.

In an attempt to better specify hypoperfused tissue, we considered functionally compromised tissue conservatively as being 1.5 standard deviations below average CBF_RH_ for each of our participants (see Table 3 for values). After establishing an individualized threshold for hypoperfusion in each of our participants, we used those CBF thresholds to (1) individually map CBF changes in perilesional tissue (extending outward from 0 mm to 12 mm in 3 mm bands, Section 4.4), (2) examine hypoperfusion in language regions of the left hemisphere (Section 4.5), and (3) link these patterns to language behavior (Section 4.6). For readers who are interested in changes within the perilesional tissue, we provide additional analyses in the Appendix that explores (at both the group- and individual- levels) CBF_LH_ in the four 3 mm perilesional bands compared to homologue (CBF_RH_) 3 mm bands (Appendix A) as well as individual intrahemispheric (CBF_LH_) differences across the four perilesional bands (Appendix B).

### 4.4. Defining Hypoperfusion within Perilesional Rings: Individualized Thresholding

In this section, we individually identified functionally affected brain tissue based on 1.5 standard deviations below each participant’s mean CBF_RH_ and compared these values across all four perilesional bands. At the group level, CBF values in the 0–3 mm band did not differ from the calculated threshold of hypoperfusion (1.5 SD < mean CBF_RH_, t (5) = −1.18, *p* = 0.15). Unsurprisingly, this replicates findings that tissue in the 0–3 mm band is dysfunctional/hypoperfused. This hypoperfused 0–3 mm band is statistically different at the group level from all the other bands (3–6 mm: t (5) = 6.69, *p* = 0.001; 6–9 mm: t (5) = 5.52, *p* = 0.001; and 9–12 mm: t (5) = 4.96, *p* = 0.002).

**Figure 3:**
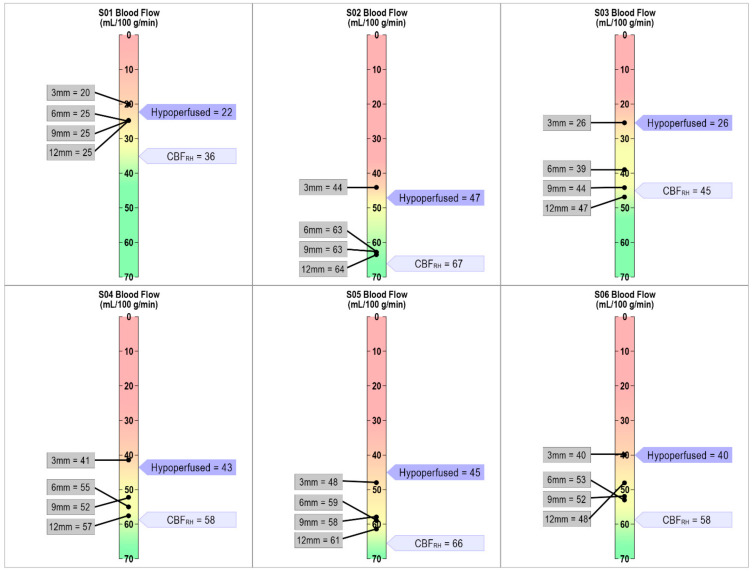
Individualized CBF threshold maps for each participant. The dark purple flag indicates when tissue is hypoperfused based on the individually defined approach of 1.5 SD below CBF_RH_. The light purple flag indicates average CBF_RH_. The gray boxes show CBF values for each of the four 3mm perilesional bands. As can be seen in the figure, the 0–3mm band is either at or below the individually defined threshold for hypoperfusion for each participant.

This landscape changed when looking at **individual patterns** of CBF in each of the perilesional bands. As can be seen in Figure 3, in the 0–3 mm band, CBF values were at or below the cutoff threshold for all participants. As we moved outward from the lesion’s rim, CBF values within the perilesional bands increased by different amounts for each participant, but overall, we saw a return toward “normal” blood flow values outside of the 0–3 mm band. We argue that our individualized thresholding provides precision in determining when perilesional tissue approximates “normal” on a case-by-case basis.

### 4.5. Identifying Hypoperfused Tissue in Language Regions

Recall that the individuals under investigation in this paper are individuals with aphasia (IWA) who have language impairments post-stroke. Throughout this paper, we emphasized the importance of looking at individualized CBF metrics for understanding brain function post-stroke, particularly in a population whose recovery is influenced by a variety of factors that can affect CBF. We demonstrated its importance in defining functionally compromised brain tissue through group versus individual-level and standard versus individualized threshold analyses.

In this section, we explore how determining hypoperfused tissue through individual thresholding better aligns with observed language-impaired behavior in IWA, as other approaches are likely to miss compromised brain regions within the left hemisphere language network. We examined mean CBF values in the remaining intact tissue of 11 left hemisphere language ROIs for each participant (see Appendix C, Table A4 for mean CBF values). Table 4 shows the percentage of tissue remaining with hypoperfused values noted in two distinct ways: (1) individual threshold calculations (1.5 SD < CBF_RH_), shaded blue and (2) standard value thresholds (<20 mL/100 g of tissue/min), indicated by red font. In doing so we discovered that individualized thresholding identified more regions of compromised tissue (see Table 4). As expected, language ROIs that had less than 10% of intact tissue remaining were mostly hypoperfused. What we want to focus attention on here is that if we were to use the standard cutoff thresholds, we would miss cases such as those found in S04, where the middle temporal pole has 100% of tissue intact but the CBF value was approximately 32 mL/100 g tissue/min. This value, it turns out, is below this participant’s 1.5 SD cutoff of their mean CBF_RH_, rendering it functionally compromised. This discrepant pattern of amount of tissue remaining and functionality can be seen across participants and ROIs only when using individualized threshold calculations (blue shading in Table 4). This suggests that evidence of structurally viable tissue alone does not translate to functionality of that region. Individualized thresholding of CBF is the only way to determine whether tissue near or far from the lesion is functionally compromised.

### 4.6. Hypoperfusion and Language Behavior

These data, as presented, lay the foundation to raise questions regarding existing attempts in the literature to link brain and language behavior. A recurring issue in the literature is the reproducibility of lesion patterns and language behavior post-stroke. Here, we argue that areas of the brain functionally impacted by stroke are often overlooked when modeling language networks. Furthermore, the way in which functionally compromised brain tissue is identified has been coarse grained at best. Below we present exploratory analyses (due to the small sample size) that looks at correlations between hypoperfused tissue (defined individually and by standard cutoffs) and language abilities. To do this, we performed two different point-biserial correlations using both our individualized metric and the standard cutoff CBF metric. In these analyses, we included hypoperfusion as a binary variable within all of our language ROIs and compared that to performance on three different language assessments, one measuring overall language severity (WAB-AQ) and two measuring basic auditory comprehension skills (BDAE-AC and WAB-AC; See Table 1 for individual scores and section “2.2. Behavioral Measures” for details on the assessments).

As a check on our language metrics, we found that both auditory comprehension (AC) subtests of the BDAE-3 and WAB-R were correlated with one another (r (5) = 0.97, *p* = 0.001). When looking at correlation results using standard cutoff values, the only areas that were correlated with AC language performance were the angular gyrus (AG) and the inferior parietal lobule (IPL) (Table 5). The WAB-AQ did not correlate with any regions of interest. However, when we performed this same analysis using our individualized hypoperfusion metrics, we found a significant relationship between multiple temporal and parietal regions and our language measures.

With the standard cutoff approach, we expected a relationship to be revealed in temporal regions since they have been implicated in auditory comprehension but did not find one [55,56,57]. The pattern in the temporal lobe did emerge when we used individualized thresholding to define hypoperfused tissue which is more in line with the literature linking auditory comprehension to regions within the temporal lobe. These patterns of results are indicative of how using individualized metrics of tissue function can provide insight into disruptions of language behavior in chronic aphasia.

**Figure 4:**
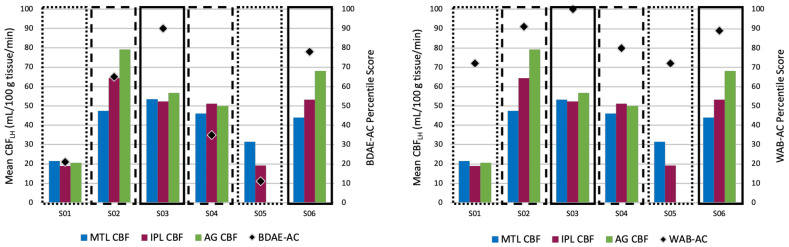
Individual CBF patterns in ROIs that were significantly correlated with the BDAE-AC (**left**) and the WAB-AC (**right**) and percentile scores. Dotted boxes represent individuals with Broca’s aphasia who performed the worst on the two auditory comprehension measures. Dashed boxes represent individuals with Broca’s aphasia who performed poorly on the BDAE-AC but not the WAB-AC. Solid boxes represent individuals with Anomic aphasia who performed well on the two auditory comprehension measures. MTL: medial temporal lobe; IPL: inferior parietal lobe; AG: angular gyrus. *Note*: S05 had a CBF value near zero in the AG, likely due to a small number of voxels not included in the lesion mask.

As a follow up to the significant correlations seen in the MTL, IPL and AG, we examined these results at the individual level (Figure 4). Interestingly, we found that the two individuals with Broca’s aphasia who performed the worst on both the BDAE-AC and the WAB-AC (S01 and S05) showed structural and functional damage in all three of these ROIs. The two individuals with Anomic aphasia who performed well on both auditory comprehension subtests (S03 and S06) showed no involvement of any of these ROIs. What we believe to be quite intriguing was that the two individuals with Broca’s aphasia (S02 and S04) who showed poor performance only on the BDAE-AC (which has a subtest that taps into semantic-conceptual knowledge, unlike the WAB-R), had structural but not functional damage in a subset of these three ROIs. While there is not enough data to interpret this pattern, it demonstrates that further investigation is warranted. Overall, these results suggest that information about both the structural and functional integrity of brain regions is important to consider for explaining language behavior outcomes.

Though the results presented in this section are only exploratory given our sample size, they do show the utility of investigating how individualized CBF metrics relate to language behavior. They also have implications for language treatment techniques, such as non-invasive brain stimulation (NIBS), that target regions of the brain that will likely respond to treatment. By using an individualized approach for defining hypoperfused tissue, both the structural and functional integrity of brain regions can be considered, which may enhance the accuracy of treatment protocols.

## 5. Discussion

The aim of the current study was to explore an alternative approach to investigating functional integrity of brain regions in chronic aphasia. In this study, we measured cerebral blood flow (CBF) in six individuals using a resting-state perfusion imaging scan protocol. With these data, we compared mean CBF in the left (CBF_LH_) and right hemispheres (CBF_RH_) at both the group and individual levels, revealing overall lower CBF values in the left compared to the right hemisphere. We also showed that lesion size alone does not determine the level of CBF found in the remaining intact tissue in the left hemisphere. In service of our goal of identifying a better way to determine whether structurally intact tissue is functionally operational, we contrasted two approaches across our participants (standard cutoff and individualized thresholds). We argued that an individualized approach provides a more accurate representation for participants when mapping out functionally compromised tissue across four different 3 mm perilesional bands. Finally, we provided preliminary evidence that this individualized approach better links brain function to language behavior by analyzing CBF values in 11 different language regions of interest (ROIs).

Hypoperfusion, or reduced CBF, is often considered to be limited to the regions within and directly surrounding the lesion in chronic aphasia, but prior research has demonstrated that regions both near and remote from the lesion can be affected by the stroke. Given the close relationship between neuronal function and CBF, disruptions of CBF can have downstream effects on behavior. In regard to aphasia, these disruptions generally occur within the language network and can adversely affect language abilities. Yet, when considering the impact of a stroke on language behavior, many research studies focus solely on relating structural damage to language impairment. However, we know that certain areas of the brain, such as perilesional tissue, can be functionally compromised despite not having any obvious structural damage. Regions such as these may contribute to language impairments that would not otherwise be predicted on the basis of structural damage alone.

Across the aphasia literature, there is a lack of clear criteria for defining perilesional tissue or defining when tissue becomes functionally compromised. Studies that investigate these factors most commonly conduct analyses at the group level, which means that important individual differences may be overshadowed by group effects. What constitutes hypoperfusion for one participant may not be as accurate for another, as CBF is moderated by many factors. While informative, these group-level analyses tend to base functionality off of whether there are significant differences between an affected region of interest and another unaffected region, often in the right hemisphere. As this may overshadow important differences at the individual level, we advocate for using an individualized approach. Additionally, standard threshold values for defining hypoperfused tissue (50–60 mL/100 g/min = normal; 12–20 mL/100 g/min = hypoperfused; <12 mL/100 g/min = necrotic) may over- or underestimate functionality. Between 25 and 50 mL/100 g/min a number of cellular changes occur that could impact function. The brain has built-in compensatory mechanisms to try to mitigate these effects, but it is possible that when CBF is within this range, brain function is altered.

Therefore, in this study, in an effort to explore individual differences in brain tissue functionality, we first compared CBF_LH_ to CBF_RH_. We saw similar patterns at both the group- and individual- level, where, unsurprisingly, CBF_LH_ was lower than CBF_RH_. Our next question was whether CBF in the remaining intact left hemisphere was impacted by lesion size. The results showed no clear relationship between lesion size and CBF, suggesting that additional factors beyond lesion size may influence CBF values in the intact left hemisphere in chronic aphasia.

After investigating overall differences between CBF_LH_ and CBF_RH_, we defined normal and hypoperfused tissue for each of our participants (see Appendix A and Appendix B for additional comparisons). To do this, we used each participant’s CBF_RH_ to determine “normal” tissue function. We then identified hypoperfused tissue by calculating 1.5 standard deviations (SD) below each participant’s mean CBF_RH_. By using the mean CBF_RH_, it allowed us to consider CBF across a wider cortical network of regions rather than just one particular region. Our individualized approach revealed that the perilesional tissue 0–3 mm from the lesion’s rim approximated 1.5 SD below mean CBF_RH_, the threshold we determined to define hypoperfused tissue. As we expanded these bands outwards, the CBF values increased in different ways across our participants but never quite reached CBF_RH_ (except for 1 participant). These differing patterns indicated that for some, tissue as close as 3–6 mm from the lesion’s border began to approximate ‘normal’ CBF function.

All of these results underscore the necessity of identifying the structural and functional integrity of brain regions when attempting to link brain and behavior. Structural lesion mapping techniques have provided important insights into this link but variability amongst participants often clouds this picture. The addition of functional integrity measures may help account for some of this variability. By individually mapping out functionally compromised brain tissue we can begin to understand how it may uniquely contribute to behavior. To zero in on this in terms of language function, we identified hypoperfusion in our 11 language ROIs based on both our individualized hypoperfusion metric as well as the standard hypoperfusion metric (<20 mL/100 g/min). Our goal was to compare the number of ROIs that emerged as hypoperfused, so we could understand the sensitivity of these two approaches. When we looked at hypoperfusion at the group level with both individualized and standard cutoff values, none of the ROIs emerged as being compromised. However, at the individual level, our individualized thresholding approach classified more ROI tissue as hypoperfused. This means that analyses at the group level would have missed multiple language regions that are functionally compromised, while standard cutoff values would have underestimated the extent of functional damage.

Exploratory correlational analyses were conducted to gauge links between hypoperfusion in language related ROIs and behavioral language patterns, as it is argued that discrepancies in the literature may be driven by a lack of consideration for how functionally compromised brain regions impact behavior. To provide assurance in our analyses, we found that all our language measures were significantly correlated with one another. When we explored the link between language and CBF patterns, we found a discrepant pattern between the two different approaches for defining hypoperfused tissue. While both approaches found links between auditory comprehension and the inferior parietal lobe and angular gyrus, only the individualized thresholding revealed links to the middle temporal lobe. These results are promising as temporal lobe damage has been strongly linked to comprehension impairments in aphasia. Here, we were looking at pure functional damage rather than structural which means that information about the functional integrity of brain regions can potentially help explain language impairment and can account for differences reported in the literature. This first step in defining functionally compromised tissue lays the groundwork for future studies to investigate structure-function relationships in language impairment and more broadly, other cognitive deficits.

## 6. Limitations of the Current Study

The goal of this exploratory study was to define an individualized metric for determining hypoperfusion in chronic aphasia and to investigate whether this metric better accounted for language impairments post-stroke. Within the aphasia literature, individual-level analyses are less common than group-level analyses. As such we provided preliminary evidence from a small sample of six individuals with chronic aphasia to advocate for the necessity of individual-level studies. Moving forward, future studies would benefit from larger sample sizes to allow for the use of more robust statistical measures that link patterns of hypoperfusion to language impairment. Additionally, in this study, our participants ranged in lesion size and aphasia severity. From our results we showed that lesion size was not related to CBF in the left hemisphere and that patterns of hypoperfusion using our individualized metric seemed to correlate with language behavior, but again because of our small sample size, it is difficult to generalize.

Finally, we acknowledge that without T2 FLAIR imaging, we are unable to confirm whether or not there are microvascular changes in the right hemisphere. Nevertheless, we do not believe that this impacts our interpretation of the performance of regions in the left hemisphere compared to the right. Recall that individualized thresholding was based on measuring whether or not CBF values fell 1.5 SD outside of mean CBF_RH._ Microvascular changes would introduce more variability which would have ultimately reduced our ability to identify areas as hypoperfused.

## 7. Conclusions

Overall, results from this study revealed important individual differences in CBF values that should be considered when mapping out functionally compromised brain tissue. These individual differences are often lost at the group level but may actually help in modeling structure-function relationships in the brain. When trying to predict language function based on structural damage alone, certain behaviors may be falsely attributed to patterns of structural damage or may be overlooked all together. In this paper, we argued the importance of uniquely defining functional impairment for every participant. Though this work can be time consuming with large sample sizes, it may better account for the variability in CBF patterns across individuals and may link to behavioral changes post-stroke. Given that numerous investigative (e.g., VLSM) and intervention (e.g., NIBS) techniques rely on the identification of intact (or impaired) brain regions, we believe that this approach can have widespread implications for theoretical modeling. Furthermore, approaching diagnosis and treatment with information about both the functional and structural integrity of the language network can help with bootstrapping recovery and developing novel approaches to treatment.

## Figures and Tables

**Table 1:** Demographic information and language assessment scores.

Participant	Sex/Age/Years Post-Stroke	Education (Years)	Aphasia Subtype ^1^	BDAE-3 ^2^ Severity (1 = Severe, 5 = Mild)	BDAE-AC ^3^ Percentile	WAB-AQ ^4^ Max = 100	WAB-AC ^5^ Percentile
S01	M/55/15	17	Broca’s	2	21	67.7	72
S02	M/67/9	20	Broca’s	3	65	82.6	91
S03	F/65/7	16	Anomic	4	90	95.8	100
S04	M/59/4	12	Broca’s	2	35	28.2 *	80
S05	M/58/5	-	Broca’s	2	11	50.8	72
S06	F/76/6	12	Anomic	3	78	88.2	89

^1^ Based on WAB-R subtypes. ^2^ BDAE-3: Boston Diagnostic Aphasia Examination—3rd edition severity rating scale (1 = severe, 5 = mild); ^3^ BDAE-AC: auditory comprehension composite (AC) score based on the BDAE-3 auditory comprehension subtests; ^4^ WAB-AQ: Western Aphasia Battery-Aphasia Quotient, measure of aphasia severity derived from the WAB-Revised (R) assessment (<50 = severe, 51–70 = moderate, >71 = mild); ^5^ WAB-AC: auditory comprehension composite (AC) score based on the WAB-R auditory comprehension subtests. * WAB-AQ score was low due to an inability to perform the repetition and overt naming tasks. Note: Education information was unavailable for S05.

**Table 2:** A summary of lesion information and brain volume for each participant.

Participant	Lesion Location	LH ^1^ Brain Volume (cc^3^)	RH ^2^ Brain Volume (cc^3^)	Lesion Volume (cc^3^)	% Lesion in LH
S01	L inferior and posterior frontal lobe w/subcortical extension; anterior, superior and middle temporal lobe; inferior, anterior and posterior parietal lobe	540.90	619.85	172.52	31.90
S02	L inferior and posterior frontal lobe w/subcortical extension; superior and middle temporal lobe; inferior and anterior parietal lobe	557.25	611.24	146.27	26.25
S03	L posterior frontal lobe with subcortical structures	426.01	517.34	23.26	5.46
S04	L medial and posterior frontal lobe; superior and middle temporal lobe; anterior parietal lobe	544.43	580.05	66.03	12.13
S05	L inferior and posterior frontal lobe w/subcortical extension; superior and middle temporal lobe; anterior, posterior, superior and inferior parietal lobe; middle and superior occipital lobe	436.17	487.67	180.60	41.41
S06	L superior temporal lobe	352.06	389.63	12.06	3.43

^1^ LH: left hemisphere; ^2^ RH: right hemisphere; cc^3^: cubic centimeters; percent (%) lesion in LH was calculated by dividing the lesion volume by the LH brain volume.

**Table 3:** CBF (mL/100 g tissue/min) values in gray matter for the whole brain, left (CBF_LH_) and right (CBF_RH_) hemispheres. Functionally compromised brain tissue (bolded) was calculated based on 1.5 standard deviations (SD) below mean CBF_RH._ A perfusion ratio was calculated by dividing CBF_LH_ by CBF_RH_ to demonstrate the extent of differences between the two hemispheres. Values < 1 indicate lower CBF_LH_ and values > 1 indicate greater CBF_LH_. * Indicates a significant difference at *p* < 0.05.

	Group-Level	S01	S02	S03	S04	S05	S06
Whole Brain CBF mean (SD)	50.97 (16.61)	30.06 (10.69)	62.66 (13.73)	44.18 (12.48)	56.44 (11.13)	58.62 (14.45)	54.42 (12.37)
Mean CBF_LH_ (SD)	46.94 (16.14)	24.29 (8.85)	58.36 (13.26)	43.14 (12.51)	54.44 (11.68)	51.18 (11.68)	50.95 (11.88)
Median CBF_LH_	47.73	23.63	60.46	43.55	57.05	52.1	51.31
Mean CBF_RH_ (SD)	54.92 (16.14)	35.71 (9.29)	66.78 (13.02)	45.23 (12.51)	58.35 (10.35)	65.56 (13.39)	57.90 (11.99)
Median CBF_RH_	57.04	34.9	68.75	43.91	60.55	67.57	60.11
**Functionally compromised CBF** **(1.5 SD below mean CBF_RH_)**	**30.71**	**21.77**	**47.25**	**26.46**	**42.83**	**45.48**	**39.91**
*t*-test results: Mean CBF_LH_ versus CBF_RH_	F(1,5) = 32.03, *p* < 0.00001 *	t(44) = −5.94, *p* < 0.00001 *	t(44) = −3.00, *p* < 0.01 *	t(44) = −0.79, *p* > 0.05	t(44) = −1.66, *p* = 0.05 *	t(44) = −5.29, *p* < 0.00001 *	t(44) = −2.76, *p* < 0.01 *
Perfusion Ratio (CBF_LH_ /CBF_RH_)	0.85	0.68	0.87	0.95	0.93	0.78	0.88

**Table 4:** A comparison of individualized and standard hypoperfusion approaches in structurally intact brain tissue. Percentages represent the amount of tissue remaining in each ROI after accounting for the lesion. Blue shading = hypoperfused based on 1.5 standard deviations below mean CBF_RH_. Red text = hypoperfused based on standard threshold values (<20 mL/100 g tissue/min). The total number of ROIs categorized as hypoperfused using each hypoperfusion metric is reported at the bottom of the table.

Language ROI	S01	S02	S03	S04	S05	S06
Pars Opercularis (BA44)	14.3%	27.8%	83.3%	92.2%	97.0%	99.9%
Pars Triangularis (BA45)	59.6%	98.9%	99.6%	100.0%	70.9%	100.0%
Pars Orbitalis (BA47)	85.7%	100.0%	100.0%	100.0%	79.5%	100.0%
Insula	3.1%	53.0%	96.8%	59.3%	70.4%	99.0%
Superior Temporal Lobe (STL)	15.6%	3.7%	100.0%	39.0%	25.9%	74.7%
Superior Temporal Pole (STP)	29.8%	93.3%	100.0%	98.0%	100.0%	100.0%
Middle Temporal Lobe (MTL)	82.1%	30.3%	100.0%	59.7%	41.9%	93.5%
Middle Temporal Pole (MTP)	44.1%	100.0%	100.0%	100.0%	100.0%	100.0%
Supramarginal Gyrus (SMG)	7.6%	30.1%	99.9%	54.6%	2.1%	51.2%
Angular Gyrus (AG)	94.8%	98.2%	100.0%	66.7%	2.9%	91.0%
Inferior Parietal Lobule (IPL)	56.9%	76.4%	100.0%	87.6%	6.4%	95.5%
Number of ROIs hypoperfused with **standard cutoffs**	3 out of 11	0 out of 11	0 out of 11	0 out of 11	3 out of 11	0 out of 11
Number of ROIs hypoperfused with **individualized cutoffs**	5 out of 11	4 out of 11	1 out of 11	4 out of 11	6 out of 11	2 out of 11

**Table 5:** A comparison of significant correlations between frontal, parietal and temporal ROIs and language measures using individual versus standard (<20 mL/100 g of tissue/min) hypoperfusion metrics. Gray shading represents significant correlations using the individualized approach not apparent with standard cutoffs. ^1^ WAB-AQ: Western Aphasia Battery-Aphasia Quotient; ^2^ WAB-AC: auditory comprehension composite (AC) score; ^3^ BDAE-AC: auditory comprehension (AC) composite score; * *p* < 0.05; ** *p* < 0.01; ^+^ *p* < 0.06; ^^^ no hypoperfusion found for any of the participants.

	Individual Cutoffs			Standard Cutoffs		
	WAB-AQ ^1^	WAB-AC ^2^	BDAE-AC ^3^	WAB-AQ ^1^	WAB-AC ^2^	BDAE-AC ^3^
WAB-AQ ^1^	1			1		
WAB-AC ^2^	0.71 ^+^	1		0.71 ^+^	1	
BDAE-AC ^3^	0.77 *	0.97 **	1	0.77 *	0.97 **	1
Pars Opercularis (BA44)	−0.26	−0.31	−0.23	−0.26	−0.31	−0.23
Pars Triangularis (BA45) ^^^	--	--	--	--	--	--
Pars Orbitalis (BA47) ^^^	--	--	--	--	--	--
Insula	−0.39	−0.14	−0.13	0.02	0.52	0.44
Superior Temporal Lobe (STL)	0.64	0.29	0.44	0	0	0
Superior Temporal Pole (STP)	0.39	0.14	0.13	−0.37	−0.22	−0.43
Middle Temporal Lobe (MTL)	0.29	0.83 *	0.82 *	0.023	0.52	0.44
Middle Temporal Pole (MTP)	0.14	0.14	0.01	0.02	0.52	0.44
Supramarginal Gyrus (SMG)	0.89 **	0.55	0.65	0.34	0.52	0.59
Angular Gyrus (AG)	0.29	0.83 *	0.82 *	0.29	0.83 *	0.82 *
Inferior Parietal Lobule (IPL)	0.29	0.83 *	0.82 *	0.29	0.83 *	0.82 *

## Data Availability

The data used to support the findings of this publication can be requested from the corresponding author upon request.

## Appendix A. Perilesional Masks: Left Hemisphere Versus Right Hemisphere

We tested overall CBF differences at the group level in each perilesional band across the two hemispheres. We conducted a 2 (hemisphere: left versus right) × 4 (perilesional bands: 3 mm, 6 mm, 9 mm, and 12 mm) ANOVA. As expected, results showed a main effect of hemisphere (F (1, 40) = 6.31, *p* = 0.02), indicating greater CBF_RH_ (M = 55.16, SD = 10.93) than CBF_LH_ (M = 46.11, SD = 13.74). There were no significant main effects for the perilesional bands (F (3, 40) = 0.43, *p* = 0.74) or the hemisphere × perilesional bands interaction (F (3, 40) = 1.42, *p* = 0.25).

This landscape changed when looking at **individual patterns** of CBF within the perilesional bands across the two hemispheres. T-test were conducted for each participant to compare each perilesional band to its right hemisphere homologue (Bonferroni corrected for multiple (4) comparisons; see Table A1 for a full list of t-statistics and *p*-values). To get a better sense of the extent of differences between each hemisphere, we also calculated a perfusion ratio (CBF_LH_/CBF_RH_). Individual results revealed different patterns as to when perilesional tissue had CBF values in the left hemisphere that were similar to the right hemisphere. Within the 3 mm perilesional band, each participant had significant differences in left versus right CBF. As we moved outward, we saw differences across participants as to *if* and *when* CBF became comparable to the right hemisphere homologue band; again, a nonsignificant difference with the right hemisphere is a measure we take to reflect functionally spared tissue. Significant differences between the left and right homologue bands persisted in the 3–6 mm band in all but 1 participant (S03, though see note for S04). Moving outward to the 6–9 mm band, differences persisted for 4 of the 6 participants. In the 9–12 mm band, differences remained for only 3 of the participants (S01, S05, S06; though see the note for S05). We note that these participants have different scaled lesions—the former two having the largest lesions of the group and the latter having the smallest lesion of the group, indicating again that lesion size does not seem to impact CBF in the intact left hemisphere.

**Table A1:** Mean CBF (mL/100 g tissue/min) values in the four left hemisphere (CBF_LH_) and four right hemisphere (CBF_RH_) perilesional bands. Paired t-test results are given for CBF_LH_ versus CBF_RH_ differences. A perfusion ratio was calculated by dividing CBF_LH_ by CBF_RH_ to demonstrate the extent of differences within perilesional bands between the two hemispheres. Values < 1 indicate lower CBF_LH_, and values > 1 indicate greater CBF_LH_. Bold values indicate that there was a significant difference in CBF values between the two hemispheres at *p* < 0.05. * indicates that the *p*-value survived Bonferroni correction at *p* < 0.0125.

	**S01**	**S02**	**S03**	**S04**	**S05**	**S06**
**Perilesion: 0–3 mm**						
Mean CBF_LH_ (SD)	20.04 (19.41)	44.47 (28.36)	25.71 (19.66)	41.30 (22.24)	47.53 (21.68)	40.22 (22.70)
Mean CBF_RH_ (SD)	41.81 (23.56)	68.00 (24.90)	49.81 (23.81)	62.83 (21.72)	64.07 (25.00)	61.23 (22.38)
*t*-test: CBF_LH_ vs. CBF_RH_	t(826) = −14.59, ***p* < 0.00001 ***	t(880) = −13.11, ***p* < 0.00001 ***	t(297) = −10.50, ***p* < 0.00001 ***	t(138) = −8.30, ***p* < 0.00001 ***	t(650) = −10.42, ***p* < 0.00001 ***	t(307) = −8.45, ***p* < 0.00001 ***
CBF_LH_/CBF_RH_ ratio	0.48	0.65	0.52	0.66	0.74	0.66
**Perilesion: 3–6 mm**						
Mean CBF_LH_ (SD)	25.21 (21.28)	62.51 (28.19)	38.86 (22.52)	55.38 (23.46)	59.24 (22.75)	53.02 (26.57)
Mean CBF_RH_ (SD)	39.13 (24.47)	67.99 (25.61)	41.00 (24.48)	59.10 (22.11)	65.32 (25.36)	63.90 (24.83)
*t*-test: CBF_LH_ vs. CBF_RH_	t(768) = −8.47, ***p* < 0.00001 ***	t(612) = −2.70, ***p* = 0.004 ***	t(273) = −0.83, *p* = 0.20	t(408) = −1.68, ***p* = 0.05**	t(644) = −3.28, ***p* = 0.0005 ***	t(247) = −3.35, ***p* = 0.0005 ***
CBF_LH_/CBF_RH_ ratio	0.64	0.92	0.95	0.94	0.91	0.83
**Perilesion: 6–9 mm**						
Mean CBF_LH_ (SD)	24.70 (18.75)	62.67 (26.97)	43.73 (23.87)	51.97 (21.40)	57.83 (20.71)	52.38 (27.69)
Mean CBF_RH_ (SD)	35.74 (21.92)	64.82 (29.21)	44.75 (23.90)	58.63 (23.69)	62.40 (24.31)	60.10 (25.54)
*t*-test: CBF_LH_ vs. CBF_RH_	t(754) = −7.48, ***p* < 0.00001 ***	t(570) = −0.91, *p* = 0.18	t(347) = −0.41, *p* = 0.34	t(451) = −3.15, ***p* = 0.0009 ***	t(601) = −2.52, ***p* = 0.006 ***	t(294) = −2.53, ***p* = 0.006 ***
CBF_LH_/CBF_RH_ ratio	0.69	0.97	0.98	0.89	0.93	0.87
**Perilesion: 9–12 mm**						
Mean CBF_LH_ (SD)	24.66 (17.72)	64.10 (27.37)	46.45 (22.87)	57.11 (22.40)	61.17 (25.37)	47.99 (21.52)
Mean CBF_RH_ (SD)	32.96 (21.49)	62.20 (29.14)	44.01 (25.63)	58.09 (23.93)	56.61 (24.05)	59.47 (25.03)
*t*-test: CBF_LH_ vs. CBF_RH_	t(768) = −5.90, ***p* < 0.00001 ***	t(589) = 0.81, *p* = 0.21	t(348) = 0.94, *p* = 0.17	t(496) = −0.47, *p* = 0.32	t(526) = 2.13, ***p* = 0.02**	t(420) = −5.10, ***p* < 0.00001 ***
CBF_LH_/CBF_RH_ ratio	0.75	1.03	1.06	0.98	1.08	0.81

As demonstrated by the perfusion ratios in Table A1, the greatest differences between CBF_LH_ and CBF_RH_ were in the 3 mm perilesional band, where CBF_LH_ was significantly lower than CBF_RH_. As perilesional bands expanded out to 6 mm and 9 mm, the perfusion ratios between left and right CBF increased such that CBF_LH_ was closer to the level of CBF_RH_. By the 12 mm band, three participants (S02, S03, and S05) had greater CBF_LH_ than CBF_RH_; this difference was only significant for S05. In summary, as the perilesional rings extended outward from the lesion’s rim, we saw variability when participants showed CBF_LH_ values that were comparable to CBF_RH_ values in the homologue region, if they did at all. This variability was not linked to lesion size as some with larger lesions showed a return to CBF_RH_ levels earlier than those with smaller lesions (e.g., S02 vs. S06).

## Appendix B. Left Hemisphere Perilesional Bands

To better understand how CBF in perilesional bands within the left hemisphere changes at the group level, we looked at the six planned comparisons shown in Table A2 (Bonferroni corrected for multiple comparisons).

**Table A2:** An illustration of the six planned comparisons across the perilesional bands.

Planned Comparisons			
	3–6 mm	6–9 mm	9–12 mm
0–3 mm	X	X	X
3–6 mm		X	X
6–9 mm			X

As shown in Table A3, only the comparison between the 0–3 mm and 9–12 mm perilesional bands yielded significant results (t (10) = −1.8, *p* = 0.047), indicating that CBF in the 9–12 mm band (M = 48.89, SD = 27.31) was significantly greater than CBF in the 0–3 mm band (M = 37.02, SD = 25.87), but this comparison did not survive multiple comparisons correction.

**Table A3:** Comparisons of CBF patterns at each perilesional band for group and individual levels. ^1^ Group-level analyses were conducted with mean CBF values across all participants. Underlined *p*-values reflect trends at the *p* < 0.06 level. Bolded values are significant at *p* < 0.05. * indicates that the *p*-value survived Bonferroni correction at *p* < 0.0083.

Perilesional Bands	Group Level ^1^	S01	S02	S03	S04	S05	S06
0–3 mm to 3–6 mm	t(10) = −1.69, *p *= 0.06	t(692) = −3.40, ***p* = 0.0004 ***	t(656) = −8.71, ***p* < 0.00001 ***	t(244) = −4.92, ***p* < 0.00001 ***	t(186) = −4.91, ***p* < 0.00001 ***	t(573) = −6.33, ***p* < 0.00001 ***	t(233) = −4.46, ***p* < 0.00001 ***
0–3 mm to 6–9 mm	t(10) = −1.74, *p *= 0.06	t(703) = −3.27, ***p* = 0.0006 ***	t(598) = −8.74, ***p* < 0.00001 ***	t(287) = −7.03, ***p* < 0.00001 ***	t(162) = −3.91, ***p* = 0.00008 ***	t(550) = −5.72, ***p* < 0.00001 ***	t(270) = −4.30, ***p* = 0.00001 ***
0–3 mm to 9–12 mm	t(10) = −1.85, ***p* = 0.05**	t(731) = −3.41, ***p* = 0.0003 ***	t(609) = −9.40, ***p* < 0.00001 ***	t(283) = −8.23, ***p* < 0.00001 ***	t(160) = −5.83, ***p* < 0.00001 ***	t(513) = −6.76, ***p* < 0.00001 ***	t(387) = −3.48, ***p* = 0.0003 ***
3–6 mm to 6–9 mm	t(10) = 0.01, *p* = 0.50	t(673) = 0.33, *p* = 0.37	t(579) = −0.07, *p* = 0.47	t(274) = −1.77, ***p* = 0.04**	t(402) = 1.56, *p =* 0.06	t(540) = 0.75, *p* = 0.23	t(263) = 0.19, *p* = 0.42
3–6 mm to 9–12 mm	t(10) = −0.15, *p* = 0.44	t(670) = 0.37, *p* = 0.36	t(586) = −0.69, *p* = 0.25	t(268) = −2.80, ***p* = 0.003 ***	t(414) = −0.80, *p* = 0.21	t(522) = −0.93, *p* = 0.18	t(222) = 1.79, ***p* = 0.04**
6–9 mm to 9–12 mm	t(10) = −0.17, *p* = 0.44	t(695) = 0.03, *p* = 0.49	t(557) = −0.62, *p* = 0.27	t(321) = −1.04, *p* = 0.15	t(485) = −2.60, ***p* = 0.005 ***	t(498) = −1.64, ***p* = 0.05**	t(258) = 1.59, *p *= 0.06

Similar to the **group level**, the same planned comparisons (Bonferroni corrected for six comparisons) were conducted at the **individual level** to compare adjacent bands to one another within the left hemisphere. All participants showed significant differences between the 0–3 mm band and all the other bands (3–6 mm, 6–9 mm, and 9–12 mm; see Table A3 for a list of t-statistics and *p*-values). Clearly, all participants have dysfunctional tissue adjacent to the lesion. Patterns began to diverge across participants as we moved outwards from the 0–3 mm band (see Figure 3 in main text). Specifically, only S03 began to show differentiation between the 3–6 mm and 6–9 mm bands. This continued to the outermost (9–12 mm) band, where we identified “normal” tissue by 9 mm outside of the lesion rim. Other participants showed different patterns. For example, S04 showed differentiation as early as the second band (3–6 mm). From these results and the group-level analyses, it is clear that participants show variable patterns of CBF within the perilesional bands, as well as across the two hemispheres. At the group level, some important patterns were detected, but when exploring the same comparisons at the individual level, more regional differences were illuminated. These differences may be important when using perilesional tissue information in designing individual treatment plans.

### Summary

To better understand CBF values surrounding the lesion, we looked at CBF in perilesional tissue. Based on prior research, we divided the tissue into four different 3 mm bands that extended 0–12 mm outside of the lesion’s border (0–3 mm, 3–6 mm, 6–9 mm, and 9–12 mm) and looked at differences in the perilesional bands between the left and right hemispheres. At the group level, there was a significant effect of hemisphere, indicating that CBF in right hemisphere perilesional bands was higher than CBF in left hemisphere perilesional bands, but there were no other significant effects or interactions. However, at the individual level, this pattern changed. There were significant differences between CBF_LH_ and CBF_RH_ for each participant in the 0–3 mm band. Within the other bands, results varied across participants, for some there were significant differences between left and right while for others there were not.

We also explored individual patterns within the left hemisphere by comparing CBF values across each perilesional band. Once again, at the group level, we found an underestimation of differences as only one comparison between the closest band to the lesion’s rim (0–3 mm) and the farthest band (9–12 mm) was found to be significantly different, though they did not survive multiple comparisons correction. As we probed these effects at the individual level, we found different patterns across participants. These differences are important to understand because they potentially indicate different patterns of neuronal function outside of lesioned tissue. All participants seemed to have reduced CBF just outside of the lesion (within the 0–3 mm band), but this landscape changed for each participant in unique ways as the perilesional band expanded further out. These patterns can help inform the extent of functional damage in intact brain tissue outside of the lesion and may implicate different regions that would not otherwise be detected when modeling brain and behavior.

## Appendix C

**Table A4:** A comparison of hypoperfused ROIs based on individualized (blue shading) and standard (red font) threshold values at both the group and individual level. The numbers in the table represent mean CBF (mL/100 g tissue/min) in that left hemisphere (LH) ROI. * values of 0 likely reflect a small number of voxels that were not captured in the original lesion mask due to their proximity to large sulci.

Language ROI (LH)	Group	S01	S02	S03	S04	S05	S06
Functionally compromised CBF (1.5 SD below mean CBF_RH_)	30.71	21.77	47.25	26.46	42.83	45.48	39.91
Pars Opercularis (BA44)	46.12	30.64	40.79	30.27	57.12	70.49	47.43
Pars Triangularis (BA45)	54.77	22.31	76.19	50.10	67.64	55.08	57.32
Pars Orbitalis (BA47)	52.38	25.98	67.44	39.85	61.50	56.79	62.75
Insula	41.36	16.55	56.07	25.27	48.78	52.07	49.46
Superior Temporal Lobe (STL)	42.89	44.20	40.35	48.72	29.94	45.00	49.13
Superior Temporal Pole (STP)	34.74	21.83	43.98	43.55	32.93	44.43	21.71
Middle Temporal Lobe (MTL)	40.70	21.62	47.57	53.40	46.18	31.45	44.00
Middle Temporal Pole (MTP)	32.65	15.64	43.12	35.06	31.82	46.67	23.58
Supramarginal Gyrus (SMG)	31.86	25.21	49.90	39.45	36.28	0.00 *	40.34
Angular Gyrus (AG)	45.78	20.65	79.13	56.83	49.95	0.00 *	68.15
Inferior Parietal Lobule (IPL)	43.22	19.00	64.30	52.26	51.19	19.25	53.31
Number of ROIs hypoperfused with **standard cutoffs**	0 out of 11	3 out of 11	0 out of 11	0 out of 11	0 out of 11	3 out of 11	0 out of 11
Number of ROIs hypoperfused with **individualized cutoffs**	0 out of 11	5 out of 11	4 out of 11	1 out of 11	4 out of 11	6 out of 11	2 out of 11
